# β-Cyclodextrin counteracts obesity in Western diet-fed mice but elicits a nephrotoxic effect

**DOI:** 10.1038/s41598-019-53890-z

**Published:** 2019-11-27

**Authors:** Angelique M. L. Scantlebery, Peter Ochodnicky, Lotte Kors, Elena Rampanelli, Loes M. Butter, Chaima El Boumashouli, Nike Claessen, Gwen J. Teske, Marius A. van den Bergh Weerman, Jaklien C. Leemans, Joris J. T. H. Roelofs, Sandrine Florquin

**Affiliations:** Department of Pathology, Academic Medical Center, University of Amsterdam, Amsterdam, North Holland The Netherlands

**Keywords:** Lipids, Drug safety

## Abstract

Obesity has become a worldwide health crisis and is associated with a plethora of comorbidities. The multi-organ effects of obesity have been linked to ectopic lipid accumulation. Thus, there is an urgent need to tackle the obesity crisis by developing effective lipid-lowering therapies. 2-hydroxypropyl-β-Cyclodextrin (2HP-β-CD) has been previously shown to reduce lysosomal cholesterol accumulation in a murine model of Niemann Pick Type C (NPC) disease. Using a murine model of Western diet-induced obesity (DIO), we report the effects of 2HP-β-CD in counteracting weight gain, expansion of adipose tissue mass and ectopic lipid accumulation. Interestingly, DIO caused intracellular storage of neutral lipids in hepatic tissues and of phospholipids in kidneys, both of which were prevented by 2HP-β-CD. Importantly, this report brings attention to the nephrotoxic effects of 2HP-β-CD: renal tubular damage, inflammation and fibrosis. These effects may be overlooked, as they are best appreciated upon assessment of renal histology.

## Introduction

Adult obesity is classified by a Body Mass Index (BMI) exceeding 30 kg/m^1,2^. For the past decade, this classification has included a measurement for abdominal adiposity since it has been linked to cardiovascular disease (CVD), due to its disturbance of metabolic regulation^2,3^. Although CVD, along with type 2 diabetes, are the most commonly known co-morbidities of obesity, it is, in fact, also linked to psychosocial, neurological, pulmonary, gastrointestinal, renal, musculoskeletal and (other) endocrine disorders^4^. The comforts of the Western lifestyle and mass production of food are seen as the driving forces behind the increased incidence of obesity. Should this trend continue, 1 out of 5 individuals worldwide are expected to be obese by the year 2030^5^. It is, therefore, understandable that obesity has become a global financial burden.

The kidney is known to be affected by both CVD and diabetes, however, obesity can lead to renal disease in the absence of either of these comorbidities^6^. Ectopic lipid accumulation is now believed to be the link between obesity and many of its comorbidities^7^. This lipid accumulation in non-adipose tissue leads to cellular damage and eventually organ dysfunction, an effect known as lipid toxicity^8^. As it pertains to the kidney, many nephropathies are also associated with intrarenal lipid accumulation, highlighting renal cell susceptibility to intracellular lipid accumulation^9–11^. Given the growing obesity epidemic and the rapidly increasing prevalence of obesity-related disorders, it is exigent that therapeutic strategies are developed to prevent obesity and the associated lipid toxicity.

We have previously observed lysosomal phospholipid accumulation in the kidneys of C57BL/6 J mice that were placed on a western diet^12^. In 2009, Davidson *et. al*, inadvertently discovered that 2-hydroxypropyl-β-Cyclodextrin (2HP-β-CD) reduced lysosomal cholesterol accumulation in neurons of mice with Niemann Pick Type C (NPC) disease^13^. NPC is a neurodegenerative disorder, that results in pervasive accumulation of unesterified cholesterol and glycosphingolipids (GSL) in late endosomes and lysosomes^14,15^. Later, Zimmer *et. al* showed that 2HP-β-CD was able to reduce the formation of atherosclerotic plaques and the accumulation of cholesterol crystals in ApoE^−/−^ mice fed a cholesterol-rich diet^16^. Cyclodextrins are a family of cyclic oligosaccharides that are clinically used to improve the bioavailability of hydrophobic drugs thanks to their lipophilic central cavity and hydrophilic outer surface. Cyclodextrins are comprised of 6, 7 or 8 dextrose units and are named α, β and γ, respectively. These three are also known as the parental cyclodextrins, as they can be chemically altered for added effects or higher efficiency. 2HP-β-CD is a derivative of β-cyclodextrin and has improved water solubility. As we have previously observed renal lysosomal lipid accumulation in obese mice fed a Western diet^12^, we hypothesized that 2HP-β-CD may be effective in preventing the sequestration of lipids within the lysosome. Furthermore, we considered that 2HP-β-CD may, indirectly, avert, not only renal lipotoxicty, but also hepatic steatosis, by antagonizing excessive intracellular lipid storage.

To this end, we performed a preventative study, in which C57BL/6 J mice were fed either regular chow, referred to as a control diet (CD), or a Western diet (WD). Within each dietary group, half of the mice received a subcutaneous injection of either saline or 20% 2HP-β-CD, throughout the experimental period. We assessed markers of obesity and lipid accumulation within the kidney and liver. We found 2HP-β-CD to be effective in the prevention of weight gain, hepatic neutral lipid accumulation and renal phospholipid accumulation in WD-fed mice. We also observed severe tubular vacuolation and osmotic diuresis in mice that received 2HP-β-CD. Finally, we highlight how the liver and kidney respond differently to the WD and hope to bring awareness to the nephrotoxic effect of 2HP-β-CD, as it may go unnoticed in studies that do not assess renal histology.

## Results

### 2HP-β-CD prevents weight gain and the accumulation of adipose tissue

**Figure 1:**
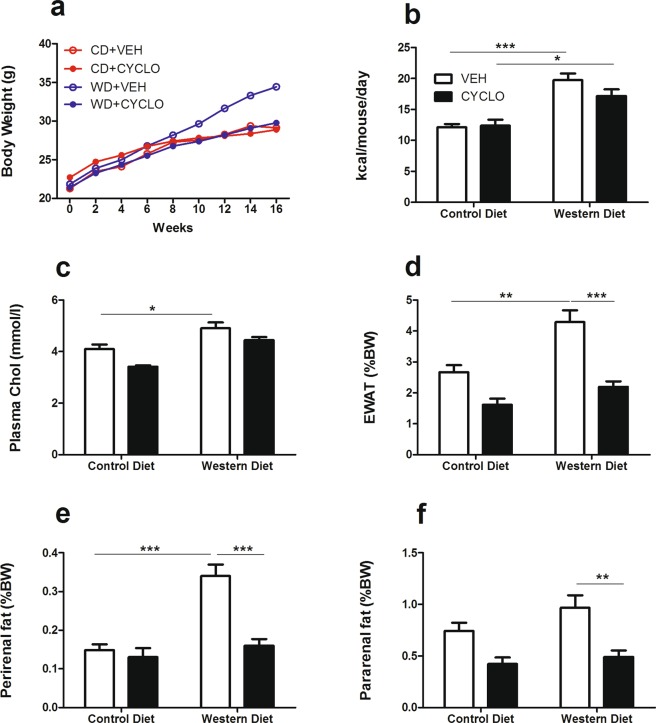
2HP-β-CD prevents weight gain and the accumulation of adipose tissue. Evaluation of weight gain, caloric intake and adipose tissue. (**a**) Body weight was monitored for 16 weeks in each experimental group. (**b**) Food intake measured as kcal/mouse/day. (**c**) Plasma cholesterol levels. (**d–f**) EWAT, perirenal and pararenal fats were weighed upon sacrifice and are shown as a percentage of total body weight. CD = control diet; VEH = vehicle; WD = western diet; CYCLO = 2HP-β-CD; kcal = kilocalorie; Chol = cholesterol; EWAT = epididymal white adipose tissue. Data shown as mean ± SEM. Statistical analysis of plasma chol, EWAT, perirenal and parerenal fat pads was performed using the One-way ANOVA test with Bonferroni post-hoc analysis. Statistical analysis of food intake was performed using the Kruskal Wallis test with Dunn’s post hoc analysis. *p > 0.05, **p > 0.01, *** p > 0.001.

Increased weight gain was observed in WD-fed mice when compared to CD-fed mice. This weight gain was prevented in WD-fed mice that received 2HP-β-CD and was comparable to that of CD-fed mice (Fig. 1a). Importantly, neither caloric intake nor plasma cholesterol concentrations were the underlying cause of this phenomenon since both measurements were similar between mice that received vehicle or cyclodextrin, within the same dietary group. These parameters were, however, increased in WD-fed mice (Fig. 1b,c).

Epididymal white adipose tissue (EWAT) and perirenal fat pads were significantly increased in WD-fed mice and administration of 2HP-β-CD inhibited this increase in adipose mass in WD-fed mice (Fig. 1d,e). Although pararenal fat was not significantly increased in WD-fed mice, a significant reduction was observed in WD-fed mice that received 2HP-β-CD (Fig. 1f).

### 2HP-β-CD prevents cholesterol and triglyceride accumulation in liver tissue

**Figure 2:**
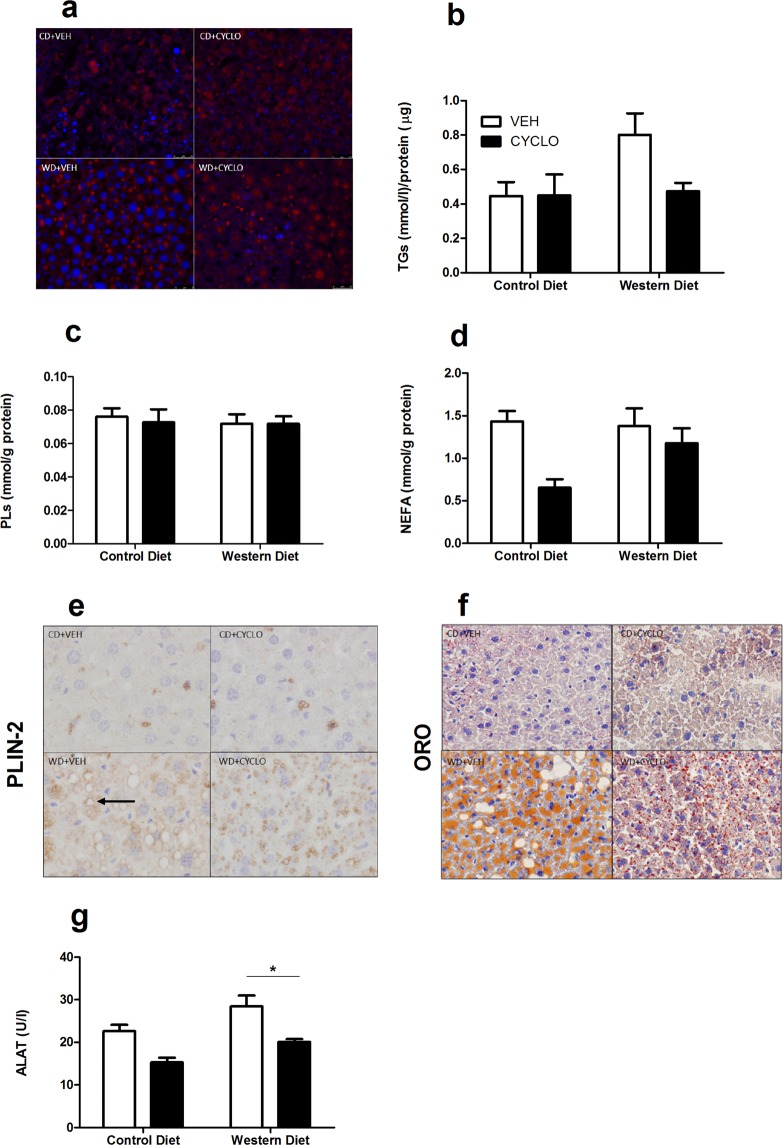
2HP-β-CD prevents cholesterol and triglyceride accumulation in liver tissue. Assessment of the effect of the WD and 2HP-β-CD on hepatic lipid accumulation. (**a**) Free cholesterol visualized in liver tissue by means of the Filipin (blue) staining, accompanied by a nuclear staining (red). (**b–d**) Triglycerides, Phospholipids and NEFA content quantified in liver homogenate. (**e**) Lipid droplets (black arrow) visualized in liver tissue with the anti-PLIN-2 antibody. (**f**)Neutral lipids visualized in liver tissue with the ORO (red) histochemical stain. (**g**) Plasma ALAT levels. TGs = Triglycerides; PLs = Phospholipids; NEFA = Non-esterified free fatty acids; PLIN-2 = Perilipin-2; ORO = Oil Red O; ALAT = Alanine aminotransferase. Data shown as mean ± SEM. Statistical analysis of TGs, PLs, NEFA and plasma ALAT measurements was performed using the One-way ANOVA test with Bonferroni post-hoc analysis. *p > 0.05.

As the liver is a major organ that is affected by obesity, we studied the accumulation of various lipids in liver tissue. WD-fed mice showed a strong accumulation of free cholesterol, visualized by Filipin staining, when compared to CD-fed mice. Conversely, WD-fed mice that received 2HP-β-CD were protected from cholesterol accumulation (Fig. 2a). Accordingly, 2HP-β-CD prevented the increase in liver triglyceride (TGs) levels (Fig. 2b). The WD did not lead to an accumulation of phospholipids nor non-esterified fatty acids (NEFA) (Fig. 2c,d). Next, we assessed the changes in the number and size of intrahepatocellular lipid droplets by Perilipin-2 (PLIN2) and Oil-Red-O (ORO) histochemical stainings. The PLIN-2 staining for lipid droplet limiting membranes showed the sporadic presence of hepatic lipid droplets in CD-fed mice. The WD led to a sharp increase in both the number and size of lipid droplets, whereas 2HP-β-CD counteracted the enlargement of these droplets (Fig. 2e). Similarly, the ORO staining showed the accumulation of neutral lipids in WD-fed mice, which was markedly decreased in mice that received 2HP-β-CD- (Fig. 2f). Although WD-feeding did not affect plasma ALAT levels, 2HP-β-CD reduced ALAT levels in WD-fed mice (Fig. 2g).

### 2HP-β-CD prevents phospholipid accumulation in renal tissue

**Figure 3:**
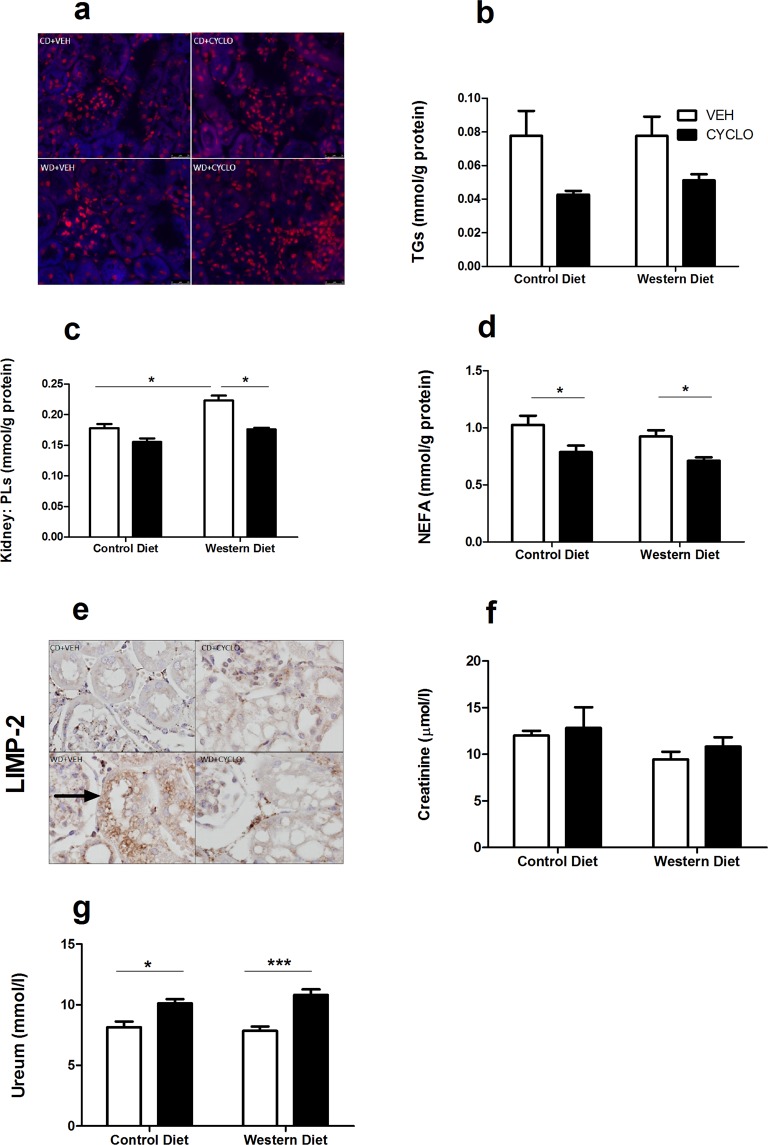
2HP-β-CD prevents phospholipid accumulation in renal tissue. Assessment of the effect of the WD and 2HP-β-CD on renal lipid accumulation. (**a**) Free cholesterol in kidneys visualized by Filipin (blue) staining, accompanied by a nuclear staining (red). (**b–d**) Triglyceride, Phospholipid and NEFA content quantified in kidney homogenate. (**e**) Lysosomes (black arrow) visualized by anti-LIMP2 staining. (**f,g**) Plasma creatinine and urea. TGs = Triglycerides; PLs = Phospholipids; NEFA = Non-esterified fatty acids; LIMP2 = Lysosome membrane protein 2. Data shown as mean ± SEM. Statistical analysis of TGs, NEFA, Creatinine and Ureum measurements was performed using the One-way ANOVA test with Bonferroni post-hoc analysis. Statistical analysis of PL measurements was performed using the Kruskal Wallis test with Dunn’s post hoc analysis.*p > 0.05, *** p > 0.001.

In sharp contrast to the liver, the kidneys of WD-fed mice did not appear to harbor more free-cholesterol (Fig. 3a). Similarly, TG levels were also unaffected by the WD, however, a trend towards reduced TG levels is observed in mice that received CD in both dietary groups (Fig. 3b). Phospholipid levels were significantly increased in WD-fed mice, and this accumulation was prevented in mice that received 2HP-β-CD (Fig. 3c). The WD did not affect NEFA levels, however, 2HP-β-CD significantly reduced NEFA levels in both dietary groups (Fig. 3d).

As we have previously shown that excessive phospholipids are mainly entrapped within lysosomes during DIO^12^, we histologically examined renal lysosomes. Enlarged lysosomal compartments, as detected by LIMP-2 positivity, were observed in the tubules of WD-fed mice and could not be detected in mice that received 2HP-β-CD (Fig. 3e). Unexpectedly, we observed cytoplasmic vacuolation of the tubular cells, exclusively in the cyclodextrin groups. Finally, we measured plasma levels of creatinine and urea as indices of renal function. Plasma creatinine levels were unaffected by the WD and 2HP-β-CD, however, plasma urea levels were significantly increased in mice that received 2HP-β-CD, irrespective of their diet (Fig. 3f,g).

### The Western diet promotes changes in expression of genes involved in lipid metabolism with opposite trends in hepatic and renal tissues

**Figure 4:**
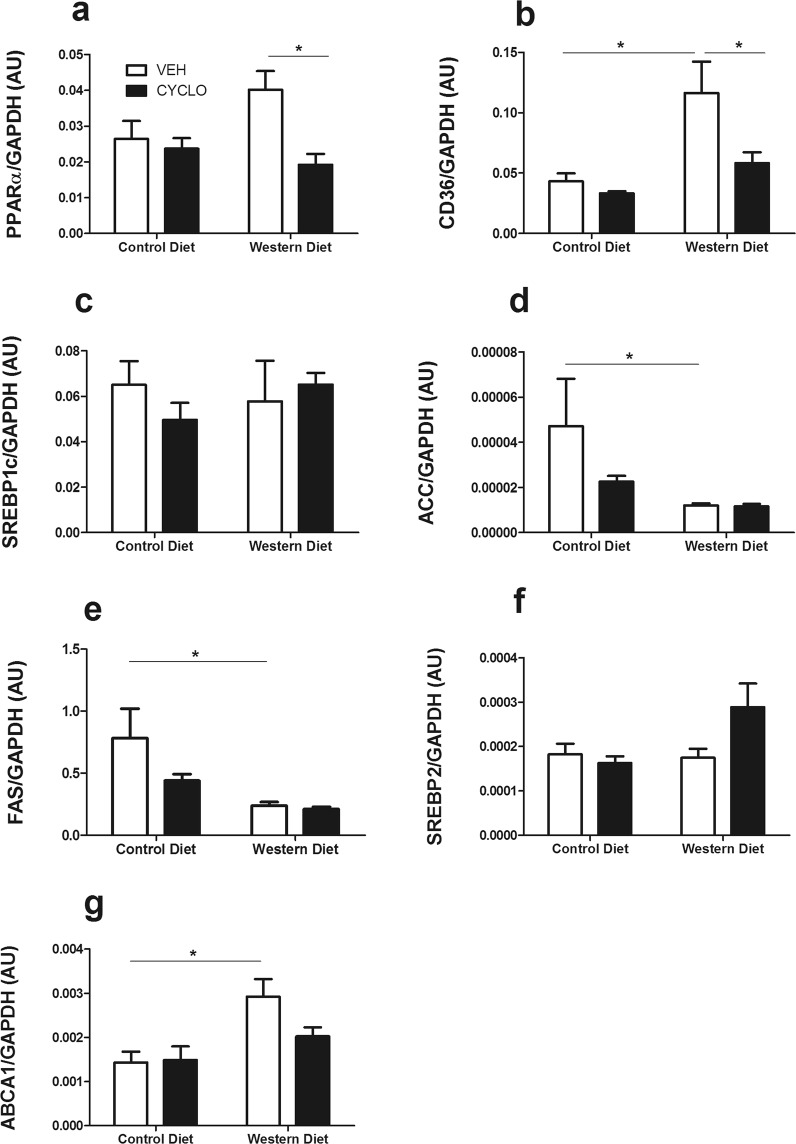
The Western diet promotes changes in the hepatic expression of genes involved in lipid metabolism. Determining the effect of the WD and 2HP-β-CD on the expression of lipid metabolism-associated genes in renal tissue. (**a,b**) Gene expression was measured for genes involved in FA uptake/utilization, (**c**) FA metabolism, (**d,e**) FA synthesis, (**f**) Cholesterol metabolism and (**g**) cholesterol efflux in liver tissue. AU = arbitrary units; *Pparα* = peroxisome proliferator activated receptor-α; *Cd36* = cluster of differentiation 36; *SREBP1c* = sterol regulatory element-binding protein 1c; *Fas* = Fatty acid synthase; *Acc* = Acetyl-CoA carboxylase; *Srebp2* = sterol regulatory element-binding protein 2; *Abca1* = ATP Binding Cassette Subfamily A Member 1. Data shown as mean ± SEM. Statistical analysis of *Pparα*, *Cd36*, *Acc*, *Srebp2* and *Abca1* levels was performed using the One-way ANOVA test with Bonferroni post-hoc analysis. Statistical analysis of *Srebp1c* and *Fas* levels was performed using the Kruskal Wallis test with Dunn’s post hoc analysis.*p > 0.05.

Next, we questioned whether 2HP-β-CD impacts the expression of genes involved in lipid metabolism, synthesis and transport. To this end, we used the following gene panel: *Pparα, Cd36, Srebp1c, Acc, Fas, Srebp2* and *Abca1*. PPARα (peroxisome proliferator activated receptor-α) is a transcription factor (TF) that promotes the upregulation of genes involved in fatty acid (FA) uptake and catabolism; CD36 is a receptor for FA uptake; SREBP1c regulates the transcription of genes involved in FA synthesis, and both ACC (acetyl-CoA carboxylase) and FAS (fatty acid synthase) are among its target genes; SREBP2 (sterol regulatory element-bind protein) is a TF that regulates cholesterol metabolism, and ABCA1 (ATP-binding cassette transporter1), a transporter responsible for the efflux of lysosomal cholesterol, is a target of SREBP2^17^. As a result of WD-feeding, gene expression of *Cd36* and *Abca1* was significantly upregulated in hepatic tissue (Fig. 4b,g), whereas *Acc* and *Fas* were inhibited (Fig. 4d,e). The gene expression of the SREBP TFs remained unaffected by DIO (Fig. 4c,f). 2HP-β-CD prevented the induction of *Pparα* and *Cd36* gene expression (Fig. 4a,b).

**Figure 5:**
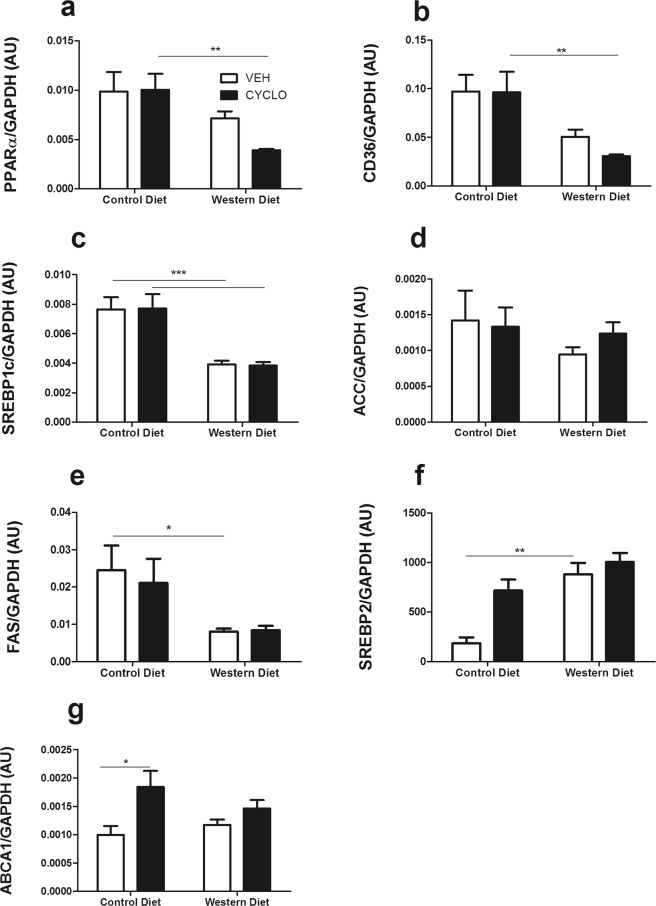
The Western diet promotes changes in the renal expression of genes involved in lipid metabolism. Determining the effect of the WD and 2HP-β-CD on the expression of lipid metabolism-associated genes in renal tissue. (**a,b**) Gene expression was measured for genes involved in FA uptake/utilization, (**c**) FA metabolism, (**d,e**) FA synthesis, (**f**) Cholesterol metabolism and (**g**) cholesterol efflux in kidney tissue. AU = arbitrary units; *Pparα* = peroxisome proliferator activated receptor-α; *Cd36* = cluster of differentiation 36; *SREBP1c* = sterol regulatory element-binding protein 1c; *Fas* = Fatty acid synthase; *Acc* = Acetyl-CoA carboxylase; *Srebp2* = sterol regulatory element-binding protein 2; *Abca1* = ATP Binding Cassette Subfamily A Member 1. Data shown as mean ± SEM. Statistical analysis of *Pparα*, *Srebp1c, Acc* and *Abca1* levels was performed using the One-way ANOVA test with Bonferroni post-hoc analysis. Statistical analysis of *Cd36*, *Fas* and *Srepb2* levels was performed using the Kruskal Wallis test with Dunn’s post hoc analysis.*p > 0.05, **p > 0.01, *** p > 0.001.

A distinct transcriptional response was observed in the kidneys. Indeed, contrary to hepatocytes, renal cells of WD-fed mice displayed lower expression of the *Pparα*, *Cd36*, *Srebp1c and Fas* genes (Fig. 5a,b,c,e), while *Srebp2* was upregulated (Fig. 5f). Expression of *Acc* and *Abca1* remained unchanged during WD (Fig. 5d,g), however, *Abca1* was the only gene affected by 2HP-β-CD, as shown by the elevated mRNA levels in CD-fed mice.

### 2HP-β-CD alters renal histology and water regulation

**Figure 6:**
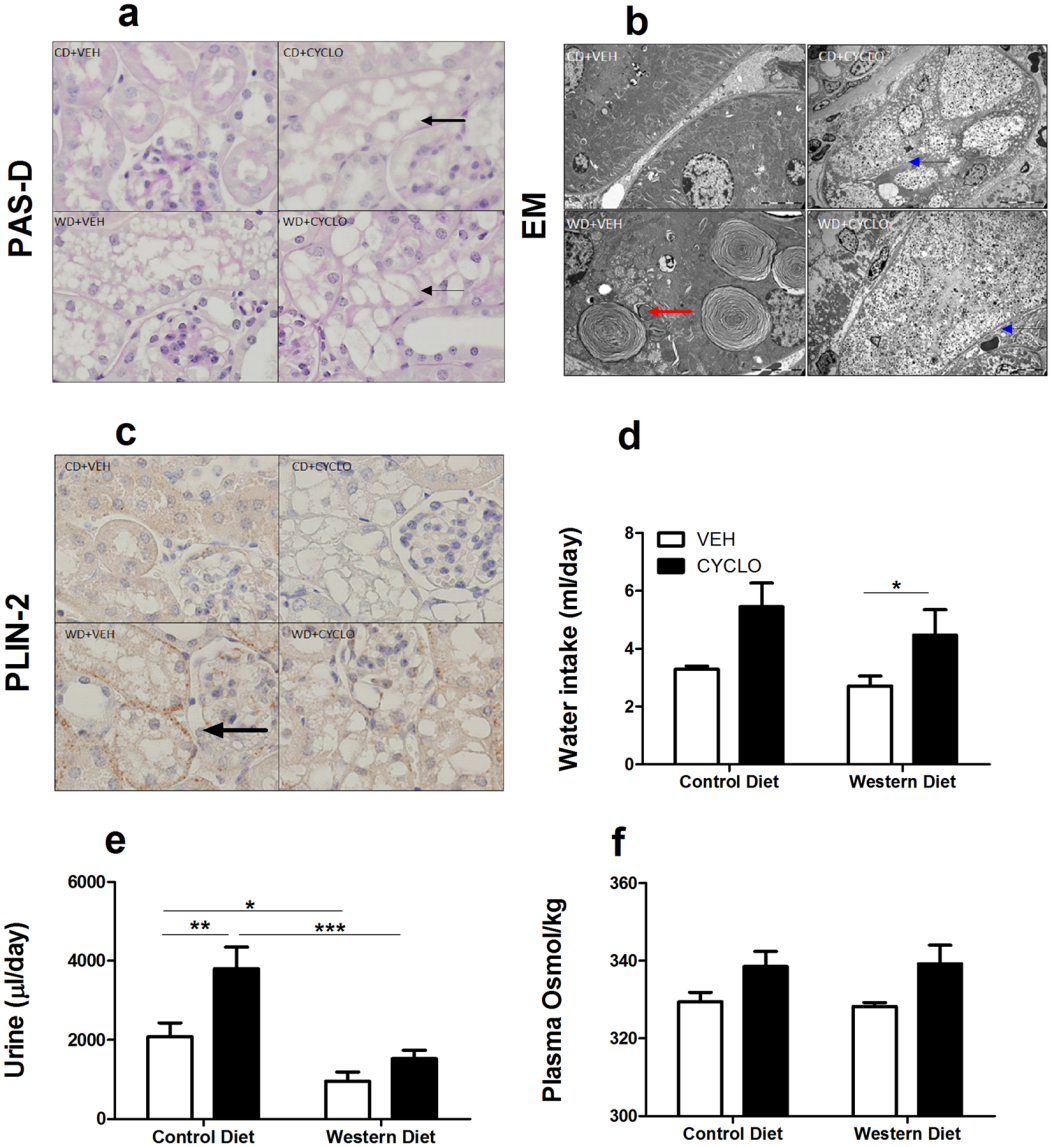
2HP-β-CD alters renal histology and water regulation. Characterization of physiological effects of 2HP-β-CD. (**a**) Renal histology visualized with the PAS-D histochemical staining. Vacuoles indicated with black arrows. (**b**) Lysosomes (red arrow) and tubular vacuoles (blue arrows) visualized by electron microscopy (EM). (**c**) Lipid droplets (black arrow) visualized in kidney tissue with the anti-PLIN-2 antibody. (**d**,**e**) Water intake and urinary output measured in week 16. (**f**) Plasma osmolarity. PAS-D = Periodic acid Schiff-Diastase; EM = electron microscopy; PLIN-2 = Perilipin-2. Data shown as mean ± SEM. Statistical analysis of water intake, urine output and plasma osmolarity was performed using the Kruskal Wallis test with Dunn’s post hoc analysis.*p > 0.05, **p > 0.01, ***p > 0.001.

As the histological examination of the renal tubules revealed extensive LIMP-2 negative cytoplasmic vacuoles, solely in mice receiving 2HP-β-CD, we deepened our interest in the possible nephrotoxic effects of prolonged cyclodextrin treatment. Histological analysis of PAS-D-stained renal sections showed small, rounded structures within the tubules of WD-fed mice, while both treated groups that received 2HP-β-CD displayed larger, non-uniformly shaped structures in the renal tubules (Fig. 6a). EM analysis revealed the presence of multilamellar bodies (MLBs) (red arrow) in WD-fed mice that did not receive 2HP-β-CD, while amorphous vacuoles (blue arrow) with a granular content were observed in mice that did receive 2HP-β-CD, irrespective of their diet (Fig. 6b). Lipid droplets were visualized on the basolateral membrane of tubular cells by PLIN-2 staining in WD-fed mice, but were far less prominent in mice that received 2HP-β-CD. The 2HP-β-CD-induced vacuoles stained negatively for PLIN2, excluding the possibility that they, too, could be lipid droplets (Fig. 6c). The WD did not affect water intake, but significantly reduced urine production (Fig. 6d,e). However, administration of 2HP-β-CD led to increased water intake in both dietary groups and increased urine volume production, in CD-fed mice (Fig. 6d,e). Despite these effects, plasma osmolality was similar amongst all groups (Fig. 6f).

### 2HP-β-CD leads to tubular damage, inflammation and early fibrosis

**Figure 7:**
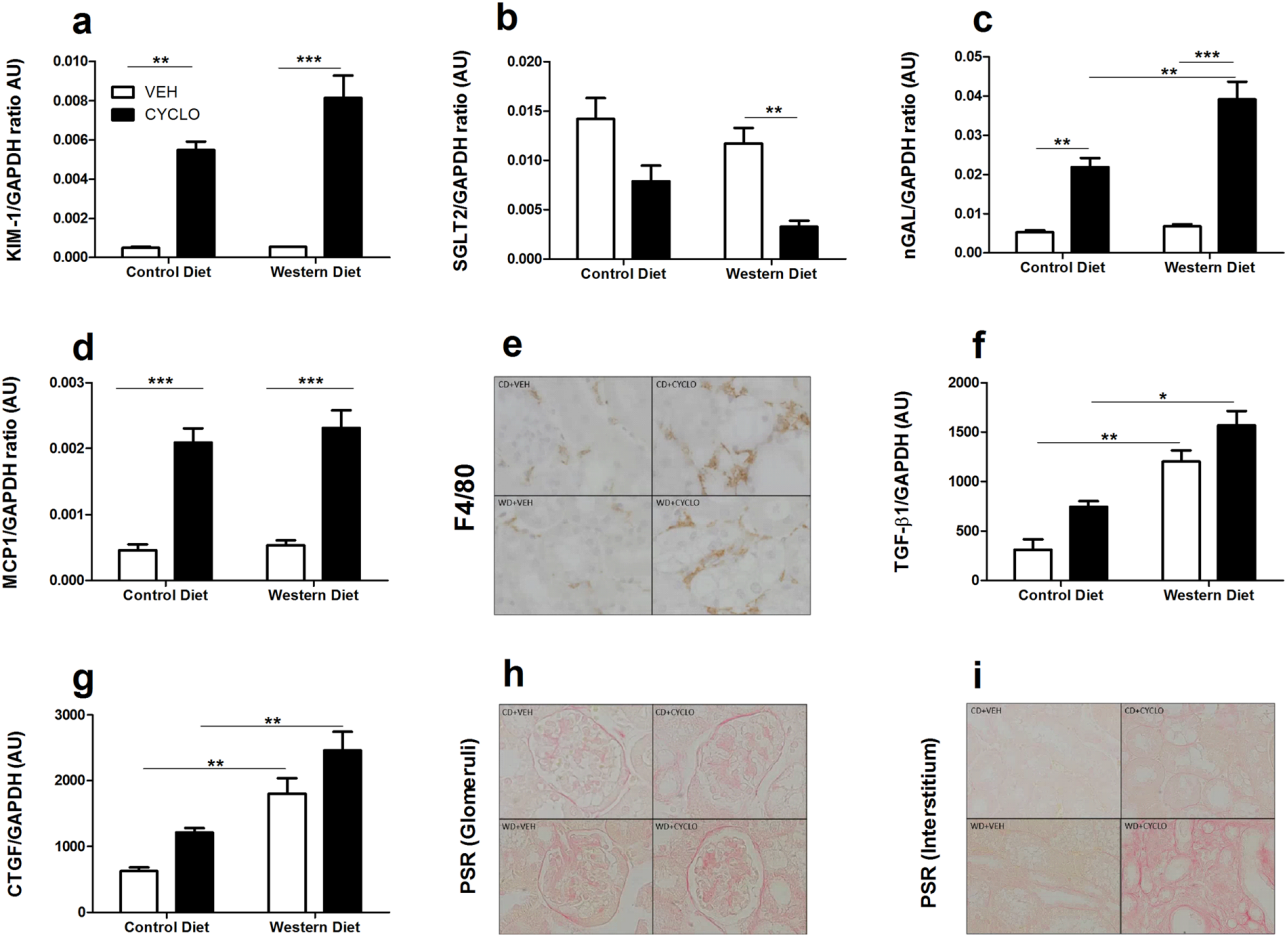
2HP-β-CD leads to tubular damage, inflammation and early fibrosis. Gene expression for damage, inflammatory and fibrotic markers were measured in renal tissue to characterize nephrotoxic effects of 2HP-β-CD. Expression of genes encoding **(a**) KIM-1, (**b**) SGLT2, (**c**) NGAL, (**d**) MCP-1. (**e**) Renal macrophage influx determined by F4/80 staining. Expression of genes encoding (**f**) TGF-β1 and (**g**) CTGF. (**h,i**) Glomerular and interstitial collagen deposition visualized with the PSR staining. AU = arbitrary units; KIM-1 = Kidney injury marker -1; SGLT2 = Sodium/Glucose Cotransporter, Member 2; nGAL = neutrophil gelatinase-associated lipocalin; F4/80 = macrophage marker; MCP-1 = Monocyte Chemoattractant Protein-1; TGF-β1 = Transforming Growth Factor Beta 1; CTGF = Connective Tissue Growth Factor; PSR = Picro Sirius Red. Data shown as mean ± SEM. Statistical analysis of *Kim-1, Lcn2* (nGAL)*, Mcp-1* and *Ctgf* expression levels was performed using the One-way ANOVA test with Bonferroni post-hoc analysis. Statistical analysis of *Sglt2* and *Tgf-β1* expression levels was performed using the Kruskal Wallis test with Dunn’s post hoc analysis.*p > 0.05, **p > 0.01, *** p > 0.001.

To further elucidate the nephrotoxic effects of 2HP-β-CD, the expression of various damage, functional, inflammatory and fibrotic markers were measured by quantitative real-time PCR (qPCR). The expression of *Kim1*, the gene encoding for kidney injury marker-1 (KIM-1), an early marker of proximal tubule injury^18^, was not affected by the WD, but was significantly induced by 2HP-β-CD (Fig. 7a). The WD did not alter the gene expression of *Sglt2*, a sodium/glucose co-transporter involved in the physiological absorptive functions of proximal tubules; however, *Sglt2* expression was decreased in mice that received 2HP-β-CD (Fig. 7b). Similarly, expression levels of *Lcn2* (nGAL), a sensitive biomarker for distal tubule injury^19^, were unaffected by the WD, but were significantly increased in mice that received 2HP-β-CD (Fig. 7c).

Since tubular injury drives inflammation^20^, we next determined the mRNA levels of monocyte chemoattractant protein-1 (MCP-1), a key chemokine for monocyte/macrophage influx. In line with the expression pattern of kidney damage markers, *Mcp1* gene expression and F4/80 staining showed a marked increase in mice subjected to 2HP-β-CD injection (Fig. 7d,e). As inflammation precedes fibrosis^20^, the expression of pro-fibrotic mediators and collagen deposition were investigated. Western diet feeding alone provoked the upregulation of transforming growth factor-β1 (*Tgfb1)* and connective tissue growth factor (*Ctgf)* gene expression and glomerular collagen I and III deposition, as determined by Picrosirius red (PSR) staining (Fig. 7f–h). Interestingly, 2HP-β-CD administration did not significantly alter *Tgf-β1 and Ctgf* expression but led to more tubulointerstitial fibrosis (Fig. 7i).

## Discussion

The rising incidence and financial burden of obesity and its comorbidities, have made it one of the most challenging health crises of the 21^st^ century. The numerous pathologies linked to obesity are attributed to ectopic lipid accumulation and the resulting organ damage or dysfunction. We aimed to prevent obesity and ectopic lipid accumulation in a DIO mouse model using 2HP-β-CD, due to its lipid-lowering properties described in NPC type C disease^13,21^ and its ability to prevent the formation of atherosclerotic plaques^16^. In particular, we focused on cyclodextrin- and WD-mediated effects on the liver and kidneys since the liver functions as a ‘logistics center’ for both dietary and *de novo* lipids and our previous studies highlight the susceptibility of renal cells to lipotoxicity^12,22^. The fat content in the WD used in this DIO model was approximately 4 times higher than that of normal chow and led to significant weight gain and ectopic lipid accumulation in the liver and kidney. In this model, 2HP-β-CD was able to prevent both obesity and ectopic lipid accumulation, but was found to cause tubular vacuolation, inflammation and fibrosis in renal tubular cells.

The liver plays an important role in lipid recycling, synthesis, packaging, storage and, most importantly, is the site at which excess cholesterol is removed through biliary excretion. Granted, once the lipid supply exceeds the liver’s processing abilities, lipid accumulation will occur. In our model of DIO, the rise in intrahepatocellular storage of neutral lipids (cholesterol esters, triacylglycerols and FA) and the enlargement of lipid droplets are indicative of hepatic steatosis, a major feature of non-alcoholic fatty liver disease (NAFLD)^23^. Nonetheless, steatosis was not accompanied by liver damage, inferring that lipid accumulation is not the sole cause of hepatic damage. In fact, liver damage ensues when NALFLD progresses to non-alcoholic steatohepatitis (NASH), a condition in which the steatosis is accompanied by inflammation, leading to fibrosis and cirrhosis^24^.

Renal disease progression was linked to dyslipidemia in the early eighties^25,26^, and through animal models, we know that proximal tubular epithelial cells (PTECs) and the glomeruli are most susceptible to lipid accumulation^27–29^. In this DIO model, the kidneys appear to be defective in the degradation of phospholipids, but not neutral lipids. Given the increased lysosomal content in proximal tubules and the observation of MLB genesis upon WD feeding, it is very likely that phospholipids are engulfed in lysosome-derived MLBs^30^.

In hepatic tissue, lipid droplets of various sizes could be observed throughout the entire liver section, while in renal tissue, small lipid droplets were specifically localized to the basolateral membrane of proximal tubules. In fact, lipid droplets were rarely observed at the corticomedullary junction. This may explain why no differences in renal neutral lipid content could be detected between CD- and WD-fed mice.

In obese mice, renal inflammation has been observed in combination with fibrosis^27,29^. In this study, however, DIO led to fibrosis, as evidenced by increased mRNA expression of fibrotic markers, but this appeared to occur in the absence of an inflammatory component. Both inflammation and fibrosis were clearly observed in mice that received 2HP-β-CD. Fibrotic markers, especially, were seen to increase even in CD-fed mice, implying that 2HP-β-CD was the driving force.

Although 2HP-β-CD has gained attention for its cholesterol depleting properties^13^, in our DIO model it was also found to mitigate triacylglycerol accumulation in the liver and kidneys, while significantly inhibiting phospholipid accumulation and MLB formation in the kidneys. Furthermore, in liver tissue, 2HP-β-CD prevented the WD-induced upregulation of *Cd36* and *Ppara*, genes involved in FA uptake and FA oxidation, respectively^31,32^. Surprisingly, in the kidneys, 2HP-β-CD had no significant effect on FA metabolism-related genes but instead upregulated the expression of *Abca1*, a transporter regulating cholesterol efflux from the lysosome to the outer cell membrane, in the CD-fed mice.

The distinct transcriptional response to the WD observed in the liver and kidneys may be linked to the type of lipids that are mainly stored in these organs. For instance, SREBP activity is suppressed by cholesterol availability^33^, which was increased in the liver but not in kidneys upon WD, with subsequent effects on the expression of SREBP-target genes. The accumulation of cholesterol by hepatocytes may have repressed SPEBP activity in the liver, whereas the renal SREBP ‘response’ to the WD, may be due to the lack of cholesterol accumulation within the kidney. However, in both organs, SREBP expression does not always align with the expression of known target genes, the best example being the WD-mediated effect on hepatic *Acc*, *Fas* and *Abca1* expression, in the absence of an effect on SREBP expression. This can be explained by the fact that the gene expression of a TF is not a reflection of its activity. SREBP genes are translated as inactive, membrane-bound precursors whose activity require proteolytic activation, nuclear translocation and the involvement of specific cofactors^33,34^. Therefore, target gene expression is arguably a better indicator of TF activity, than the expression of the TF itself.

Lastly, this report shows the 2HP-β-CD-mediated nephrotoxicity characterized by tubular vacuolation, induction of biomarkers of proximal and distal tubular damage, macrophage influx and tubulointerstitial fibrosis. Collagen deposition in the glomerular compartment was not worsened by 2HP-β-CD, suggesting that the susceptibility to damage/fibrosis differs per renal compartment. Furthermore, focal fibrosis seemed to result from the combination of both the WD and 2-HP-CD, indicating the need of a ‘double hit’. The large vacuoles within proximal tubular cells were neither identified as lipid droplets nor MLBs. This was somewhat surprising as MLBs can form in response to cationic amphiphilic drugs (CADs), which, like cyclodextrins, contain both hydrophobic and hydrophilic regions and are thought to form drug-lipid complexes resistant to lysosomal digestion^35^.

As 2-HP-CD is excreted via the urine and therefore reaches high concentrations in the renal tubules, it is plausible that it is sequestered in proximal tubules. Lysosomal enlargement in renal tubules has been observed as a common response to the excretion of highly concentrated osmotic agents, such as glucose. A study in rats reports observing ‘acicular microcrystals embedded in the lysosomal matrix’ in the proximal tubules of cyclodextrin-treated animals^36^. We were not able to confirm whether the vacuoles observed in mice that received 2-HP-CD are of lysosomal origin by means of stainings (data not shown). However, we are aware of the fact that the enlargement of the vacuoles may have distorted the epitopes required for immunohistochemical identification.

2HP-β-CD is a modified version of β-cyclodextrin that is suggested to improve water solubility, while reducing toxicity. Toxicology reports have described nephrotoxic effects of 2HP-β-CD. These effects are observed, histologically, as vacuolation of the renal tubules. Thus far, this effect has not been reported to effect renal function. Due to its chemical structure, 2-HP-CD is expected to have an osmotic effect and lead to osmotic diuresis. In fact, patents have been developed for the use of cyclodextrin and/or derivatives thereof to improve diuresis^37^. We observed this effect most prominently in CD-fed mice that received 2-HP-CD, where both water intake and urinary excretion were significantly increased. Osmotic diuresis occurs when the accumulation of a substance that cannot be reabsorbed inhibits water reabsorption and hence increases urinary excretion^38^. We can infer that tubular cells absorbed 2HP-β-CD molecules, together with water, giving rise to these enlarged vacuoles.

Based on its efficacy in the prevention of lipid accumulation, but taking into consideration the nephrotoxic effects of 2-HP-CD, we propose that new guidelines be developed for the administration of 2-HP-CD therapy. These guidelines should include 2 main aspects; 1: assessment of renal function prior to and following the administration of 2-HP-CD, and 2: incorporation of a patient-tailored, on/off regimen to maximize the lipid-lowering effects of 2-HP-CD, while dampening its nephrotoxic actions. This is based on a toxicology study in rats that received a daily dose of 400 mg/kg 2-HP-CD for 3 months, in which it was reported that the nephrotoxic effects of 2-HP-CD could no longer be observed when treatment was halted for 1 month^39^. The nephrotoxicity observed in our study did not lead to renal dysfunction within the 4-month period, however, the treatment of an obese patient is likely to require a longer treatment time. Particularly, renal function should be monitored in 2-HP-CD-treated patients with a history of renal insufficiency, as they may be more susceptible to 2-HP-CD-mediated nephrotoxic effects. Finally, other models that study the effect of 2-HP-CD on impaired lipid homeostasis but do not specifically assess renal histology, are likely to miss this nephrotoxic effect.

In our DIO model, we have shown that the liver and kidney respond differently to lipid overload. This is most likely linked to their natural roles in lipid metabolism. The WD resulted in the accumulation of lipid droplets in both the liver and kidney, but phospholipidosis (intralysosomal accumulation of phospholipids) was observed exclusively in the kidneys. We have shown that 2-HP-CD is effective in preventing weight gain, lowering plasma cholesterol levels and reducing the accumulation of both polar (phospholipids) and nonpolar (TG/cholesterol) lipids. In addition, 2-HP-CD led to extensive tubular vacuolation and damage, but did not result in renal dysfunction. We encourage those that are, or will be, administering 2-HP-CD to be aware of this effect and to consider the exploration of alternative treatment regimens to alleviate this toxicity.

## Materials and Methods

### Mice

Male wildtype (WT) C57BL/6 mice, approximately 6 weeks old, weighing 15–20 grams (Charles River) were used. Animals were housed in the animal care facilities of the Academic Medical Center (University of Amsterdam), in accordance with national guidelines and given *ad libitum* access to both food and water, except during one fasting period, just prior to sacrifice. Mice were housed in enriched cage systems with 2–6 mice per cage. A daily health monitoring report was filled out by animal care staff. This includes a scoring system for animal discomfort, overall health, responsiveness, physical movement, body weight stability, fur condition, signs of fighting/aggression (especially among males), appetite and water consumption. At the animal facility, all rooms operate as barriers with 100% fresh, HEPA-filtered air (under positive pressure in most areas). The movement of persons, equipment and samples follows pre-defined traffic patterns to prevent clean and dirty supplies from crossing paths. Environmental conditions such as temperature, relative humidity, air flow, light intensity and management of light and dark periods were monitored daily. These were in accordance with the criteria specified by the American Association for Laboratory Animal Science (AALAS).

Group sizes were calculated based on a power analysis and possible unresponsiveness (≈30%) to the western diet. Mice were allocated to treatment or control groups by animal caretakers which ensured no *a priori* knowledge of group assignment and prevented subjective experimenter bias. Animal welfare was further monitored by the experimenter by means of weekly body weight measurements. All experiments were approved by the Animal Care and Use Committee of the University of Amsterdam.

### Diet-induced obesity model

Mice were divided into 4 experimental groups: Control diet + vehicle (CD + VEH), Control diet + β-cyclodextrin (CD + Cyclo), Western diet + vehicle (WD + VEH) and Western diet + β-cyclodextrin (WD + Cyclo). After one week of acclimatization, mice were either fed a control diet or a western diet. Control-diet-fed mice (n = 7 per group) received standard chow (Arie Blok AMC RD Western Diet Control 4021.84). This diet contained 19% protein, 11% fat and 70% carbohydrate, based on the caloric intake percentage(%kcal). Western-diet-fed mice (n = 12 per group) received enriched chow, containing 17% protein, 40% carbohydrates and 43% fat, based on %kcal, as well as 0.15% cholesterol (Arie Blok AMC RD Western diet 4021.83). Mice were kept on their respective diets for 16 weeks. After starting the diet (week1), mice either received twice weekly subcutaneous injections of 20% (w/v) 2-hydroxypropyl-β-cyclodextrin, 4000 mg/kg body weight, in a maximum injection volume of 500 μl or a physiological salt solution.

Food intake was recorded throughout the study while water intake was recorded for 3 weeks during the study. At weeks 0, 8 and 16, mice were placed in metabolic cages for the collection of 24-hour urine. Mice were fasted for 4 hours prior to sacrifice. Blood was collected by means of cardiac puncture, under general anesthesia (4% isoflurane/100% oxygen), followed by cervical dislocation. Blood collected upon sacrifice was spun down for 10 minutes. The supernatant was stored in a new Eppendorf vial, and this plasma was stored at −80 °C until further analyses could be performed.

The kidneys, liver, epididymal white adipose tissue (EWAT), renal fat (peri- and pararenal fat), intercapsular brown adipose tissue (BAT) and liver were collected and weighed. Kidneys, liver and epididymal fat pads were divided, allowing one section to be snap frozen in liquid nitrogen while the other was fixed in formalin prior to paraffin embedding.

### Plasma measurements (Plasma Chol + TG, Creat, Ureum and ALAT)

Plasma cholesterol was measured in the automated Roche cobas c502 Chemistry Analyser by means of an enzymatic assay. Plasma triglycerides were quantified by a enzymatic-colormetric assay performed in the Roche cobas c702 Chemistry Analyzer. Plasma creatinine, ureum and ALAT (Alanine aminotransferase) were all measured on the Roche cobas c702 Chemistry Analyzer, by means of enzymatic-colormetric, kinetic-enzymatic and spectrophotometric assays, respectively. All assays were performed at the Laboratory for General Clinical Chemistry (LAKC) at the Academic Medical Center of Amsterdam.

### Lipid measurements (in liver homogenate)

Liver homogenates were prepared by mechanically disrupting 20–30 grams of snap-frozen tissue in Greenberger lysis buffer(150 mM NaCl, 15 mM TRIS, 1 mM MgCl.6H_2_O, 1 mM, CaCl2.2HP_2_O, 1% Triton), set to pH 4 with HCL, containing a protease inhibitor cocktail (Sigma Aldrich). Samples were lysed for 30 minutes on ice, after which they were spun down for 10 minutes at 4 °C. Supernatants were stored in new Eppendorf tubes. Protein concentration was measured by means of the Bicinchoninic Acid Assay (BCA) (Thermo Fisher Scientific, Waltham, MA, USA) to allow for protein correction of the lipid assays.

Liver triglyceride levels were measured with the Triglycerides GPO Method kit (80019 Biolabo, Fr). Free (also referred to as total cholesterol) and esterified cholesterol (cholesterol esters) were measured by the Total cholesterol assay kit (fluorometric) (Cell Ciolabs Inc., US). Phospholipids were measured by the Phospholipids Colormetric Enzymatic method (99105 Biolabo, Fr) and non-esterified free fatty acids were measured with the NEFA FS (Diagnostic Systems, Holzheim, Germany).

### Histochemical stainings

Liver and renal tissues were stained for neutral lipids (Oil-Red-O). Frozen sections (4 μM-thick) were allowed to dry at room temperature followed by a 2 minute fixation in Formol-Venofundin. Slides were then incubated in the ORO (60%) solution for 25 minutes and Hematoxylin solution (50%, Klinipath) was used as a nuclear stain. Slides were sealed with glycerol-gelatine.

Renal tissue was also stained with PAS-D to observe the general histology. (FFPE) tissue was deparaffinized and rehydrated. Tissue sections were incubated in a 0.25% amylase solution (Sigma Aldrich), followed by an incubation in 1% periodic acid (Merck) and were finally incubated in Schiff’s reagent (Merck). Hematoxylin solution (50%, Klinipath) was used as a nuclear stain, after which sections were dehydrated and sealed with Pertex.

Renal tissues were further stained for collagen deposition by means of the Picro Sirius Red staining. This staining was performed at the histological lab of the pathology department, using a standard protocol.

### Immunohistochemical stainings

FFPE liver and renal tissues were stained for macrophages (F4/80), lipid droplets (PLIN-2), lysosomes (LIMP-2) and lipid peroxidation (4-HNE). FFPE tissue was deparaffinized and rehydrated. Sections were incubated in 0.3% H_2_O_2_ in methanol to block endogenous peroxidase and boiled in 0.01 M pH 6.0 citrate buffer for epitope retrieval.

For macrophage detection, tissue sections were incubated with *rat IgG2b-anti-F4/80* (eBioscience) primary antibody followed by *rabbit-anti-rat* (Dako) secondary antibody.

For the detection of lipid droplets, tissue sections were incubated with *guinea pig-anti PLIN-2* (Progen Biotechnik) primary antibody followed by the *rabbit-anti-guinea pig* (Thermo Scientific).

For the detection of lysosomes, tissue sections were incubated in *rabbit-anti-mouse LIMP-2* (LS-B305, LifeSpan Biosciences).

For the detection of lipid peroxidation products, tissue sections were incubated with *rabbit-anti-mouse 4-HNE* mAb primary antibody (Abcam). All sections were incubated with a tertiary, peroxidase-labeled antibody (Powervision poly HRP-anti rabbit, Immunologic, Duiven, The Netherlands) prior to visualization. PLIN-2- and 4-HNE-stained sections were visualized with the DAB Plus system (Dako). F4/80- and LIMP-2-stained sections were visualized with 1% H_2_O_2_ and DAB (Sigma-Aldrich) in 0.05 M Tris-HCL. Hematoxylin solution (50%, Klinipath) was used as a standard nuclear stain, after which sections were dehydrated and sealed with Pertex. All primary antibody incubations were performed overnight at 4 °C. All secondary and tertiary antibody incubations were performed for 30 minutes at RT.

### Real time-qPCR

Total RNA was isolated from 300µm-thick frozen renal sections using TRIzol (Invitrogen, Breda, The Netherlands) reagents. RNA isolation was performed according to the manufacturer’s instructions. Complementary DNA was generated following a standard protocol. Briefly, oligo-dT primers were ligated to the RNA for 10 minutes at 72 °C, followed by a 60 minute polymerization at 37 °C with M-MLV reverse transcriptase (Promega). Real time cDNA quantification was performed on the Roche LightCycler 480 (Roche Diagnostics) using SensiFAST SYBR NO ROX kit (Bioline). Gene expression in liver cDNA samples was normalized against Glyceraldehyde 3-phosphate dehydrogenase (GAPDH) expression, while that of renal samples were normalized against hypoxanthine guanine phosphoribosyltransferase (HPRT) expression. Analysis was performed using the LinRegPCR 12.4 software. Expression of lipid-associated genes (*Pparα, Cd36, Srebp1c, Aaa, Fas, Srebp2 and Abca1*) were analysed in both liver and renal cDNA samples. Expression of *Kim-1, Sglt2, Lcn2 (nGAL), Mcp-1, Tgf-β1 and Ctgf* were analysed in renal cDNA samples. Primer sequences are listed in Supplementary Table S1.

### EM analysis (kidney)

A thin transverse slice of kidney tissue was fixed in Karnofsky’s fluid and prepared at the electron microscopy department at the Academic Medical Center in Amsterdam. Photos were taken of the lipid droplets and tubular vacuolization to visualize their contents.

### Statistical analysis

Data were anlaysed using Graphpad Prism 5 software and are shown as mean ± SEM. Data was tested for normality using the D’Agostino & Pearson omnibus normality tests. Statistical analysis was then performed using the Kruskal Wallis and Dunn’s post hoc analysis or the One-way ANOVA with Bonferroni post-hoc analysis. The Grubbs test for outliers was used for the exclusion of sample data.

## Supplementary information

Supplementary Table S1

## Data Availability

The data used to support the findings of this study are included within the article.
