# Supplementary material for: β-Cyclodextrin counteracts obesity in Western diet-fed mice but elicits a nephrotoxic effect
Source: Sci Rep. 2019 Nov 27;9:17633. doi: 10.1038/s41598-019-53890-z (PMC6881402; doi:10.1038/s41598-019-53890-z)
Supplement: Supplementary file 1 — Supplementary Table S1 [file 41598_2019_53890_MOESM1_ESM.pdf]

β-Cyclodextrin counteracts obesity in Western diet-fed mice but elicits a nephrotoxic effect

Angelique M.L. Scantlebery*, Peter Ochodnicky, Lotte Kors, Elena Rampanelli, Loes M. Butter, Chaima El Boumashouli, Nike Claessen, Gwen J. Teske, Marius A. van den Bergh Weerman, Jaklien C. Leemans, Joris J.T.H. Roelofs, Sandrine Florquin

Gene	Forward	Reverse
PPARα	CACGCATGTGAAGGCTGTAA	GCTCCGATCACACTTGTCG
CD36	GGGATTGGAGTGGTGATGTTTGTTGCT	CGGGTTTACCAAAGATGTAGCCAGTGT
SREBP1c	GGCACTAAGTGCCCTCAACCT	GCCACATAGATCTCTGCCAGTGT
ACC	CCCAGCAGAATAAAGCTACTTTGG	TCCTTTTGTGCAACTAGGAACGT
FAS	CCTGGATAGCATTCCGAACCT	AGCACATCTCGAAGGCTACACA
SREBP2	CAAGTCTGGCGTTCTGAGGAA	ATGTTCTCCTGGCGCAGCT
ABCA1	CGTTTCCGGGAAGTGTCCTA	GCTAGAGATGACAAGGAGGATGGA
KIM-1	TGGTTGCCTTCCGTGTCTCT	TCAGCTCGGGAATGCACAA
SGLT2	GGCACAGTTGGTGGCTACTT	AGAGCGCATTCCACTCAAAT
nGAL	GCCTCAAGGACGACAACATC	CTGAACCATTGGGTCTCTGC
MCP-1	CATCCACGTGTTGGCTCA	GATCATCTTGCTGGTGAATGAGT
TGF-β1	ATACGCCTGAGTGGCTGTCT	TAATACGACTCACTATAGGGGCTTGCCACCCACGTAGTAAG
CTGF	GACTCAGCCAGATCCACTCC	TCTGTAATCGCAGCTCACTC
GAPDH	TGCTCACATTAAGGGTGGTG	GAGGCTGGGATGATGTTCTG
HPRT	TCCTCCTCAGACCGCTTTT	CCTGGTTCATCATCGCTAATC

Supplementary Table S1: Table of qPCR primer sequences
